# Intestinal Parasitic Infections and the Associated Risk Factors Among Malnourished Children Attending Bele Gesgar Hospital in Oromia Region, Ethiopia

**DOI:** 10.1155/japr/5295102

**Published:** 2025-04-10

**Authors:** Zewdineh Firdu, Dejene Amente

**Affiliations:** Department of Biology, Madda Walabu University, Bale-Robe, Ethiopia

**Keywords:** Bele Gesgar hospital, intestinal parasitic infection, malnourished children, risk factors

## Abstract

**Introduction:** Human intestinal parasites are identified as causes of morbidity and mortality throughout the world, particularly in underdeveloped countries.

**Objective:** This study was conducted to assess the status of intestinal parasitic infections (IPIs) among malnourished children attending Bele Gesgar Primary Hospital and investigate the possible risk factors of infections.

**Materials and Methods:** A case-control study design was employed in this study. The parasites were detected using a wet mount, formalin–ether concentration, and modified Ziehl–Neelsen methods.

**Results:** There were 422 children under the age ≤ 14 who took part in the study. The overall prevalence of IPIs was 31.75% (134/422) among sampled children, 44.07% in malnourished, and 19.43% in well nourished. Among malnourished children, the most prevalent parasites were *Entamoeba histolytica/dispar* (11.37%), followed by *Giardia lamblia* (9.48%) and *Ascaris lumbricoides* (3.79%). The highest protozoal and parasite (helminths) infections were detected in the age groups below 5 years (46.24%) and the lowest in the age group of 11–14 (13.98%). Having no toilet (*aOR* = 3.541; *p* = 0.023), not handwashing after toilet (aOR = 3.074; *p* = 0.010), having contact with animals (aOR = 0.095; *p* = 0.001), and playing with mud and soil (aOR = 13.210; *p* = 0.001) were found as significant risk factors of parasitic infection among the malnourished children according to multivariate logistic regression analysis.

**Conclusion:** In general, this study indicated that the status of parasite infections in children showed variation based on age and sex. In addition, not toilet usage, not washing hands with soap after toilet, having contact with animals, and playing with mud and soil were significant risk factors (*p* < 0.05) for IPIs in malnourished children.

## 1. Introduction

The term parasite is defined as an organism that finds its ecological niche in another organism, and they are the causes of morbidity and mortality throughout the world, particularly in developing countries. According to Sitotaw and Shiferaw [1], the most common intestinal protozoan parasites in children are *Entamoeba histolytica*, *Giardia lamblia*, and *Cryptosporidium* species. In addition, there are intestinal helminth parasites that are known as geo-helminths and soil-transmitted helminths, namely, *Ascaris lumbricoides*, *Trichuris trichiura*, *Ancylostoma duodenale*, and *Necator americanus*. As stated by the WHO, about 24% (1.5 billion) of the world's people have been infected with intestinal parasitic infection, mainly hookworms, *Ascaris lumbricoides*, and *Trichuris trichiura*, and the prevalence of intestinal parasitic infection surpasses 50% in several areas of sub-Saharan Africa [2]. In Ethiopia, the overall status of parasitic infection was 46.88% in children with diarrheal disease. The most prevalent protozoa, *Giardia lamblia* (9.38%) and *Entamoeba histolytica/dispar* (6.25%), were observed with the highest infection rates in the age groups that varied from 6 to 10 years old [3]. Infections with IPs are not only responsible for morbidity and mortality, but they also cause nutritional problems (such as stunted growth, low vitamin A, iron deficiency anemia, weight loss, and chronic blood loss) and compromise psychological and social well-being. In addition, Feleke et al. [4] described that mental developmental impairment is also another public health challenge of IPIs, particularly in children (such as impaired growth, decreased school attendance, cognitive impairment, decreased educational achievement, and adult productivity).

According to the WHO report, the term malnutrition generally refers to both undernutrition and overnutrition, and anthropometric indicators (AI) that are commonly used to measure undernutrition in a population are stunting, wasting, and underweight [5]. Worldwide, the prevalence of undernutrition is great, and its consequence in terms of public health is alarming. In 2016, as WHO reported, it is estimated that 178 million children are undernourished, 20 million children are suffering from the most severe form of undernutrition, and 3.5–5 million annual deaths are occurring among children aged 6–59 months [6]. One form of malnutrition that is currently a global problem is stunting. Stunting is an impact that occurs due to chronic malnutrition and is a major problem for children in rural areas who experience developmental disabilities [7]. It has also resulted in the loss of adult height by 3%. Subsequently, childhood stunting might end up with a 1.4% loss in productivity [8]. Several studies have been conducted in Ethiopia to assess the prevalence of IPIs in children but failed to associate them with undernutrition [1, 9, 10]. Moreover, studies have found relationships between intestinal parasites and malnutrition in children [11, 12]. There is also limited evidence of the magnitude of undernutrition among children in the Bele Gesgar area. Therefore, this study was carried out to identify the recent burden of intestinal parasitic infection and its associated risk factors among malnourished children in Bele Gesgar Primary Hospital.

## 2. Materials and Methods

### 2.1. Description of the Study Area

This study was conducted at Bele Gesgar Primary Hospital, which is found in Bele Town. Bele is found in Oromia Regional State, Arsi Zone. This town is 272 km away from Addis Ababa City in the southeastern direction and 147 km away from Asella Town, the capital city of the Arsi Zone, in the northeastern direction. The average mean annual temperature of the district is 21°C, and the annual rainfall ranges from 800 to 1200 mm. The rainfall pattern is bimodal, which is the short rainy season (Belg season from March to April) and the long summer rainy season (Meher season from June to September) [13].

### 2.2. Research Design and Study Population

A case-control research design was used to assess intestinal parasitic infections and associated risk factors among malnourished children attending Bele Primary Hospital between February 2022 and July 2022. All the study participants were children aged below 14 visiting the hospital.

### 2.3. Sample Size Determination and Sampling Technique

A random sampling technique was employed to get data on intestinal protozoal and parasitic (helminths) infection and associated risk factors among malnourished children in Bele Gesgar Primary Hospital. Mothers/caregivers of the children were selected as principal respondents in the study for those children who were unable to give their details. In addition, health personnel were involved in gathering general information about IPIs and associated risk factors through interviews. The sample size of the study participants was determined using the sample size determination formula of the single population [14]:

 $$\begin{array}{c} N=\frac{Z\alpha /2p\left(1-p\right)}{d^{2}}, \end{array}$$

where *N* represents the total sample size, *p* represents the prevalence rate (50%) of parasites among children at Bele Gesgar Primary Hospital, *Zα*/2 represents the standard normal variable (1.96), and *d* represents the margin of sampling error (5%). Adding a 10% nonresponse rate, the total sample size was determined to be 422.

### 2.4. Inclusion and Exclusion Criteria

In this study, children aged < 14 years visiting Bele Gesgar Primary Hospital as patients were included, whereas those who received medication for any protozoal and parasitic (helminths) infection before 3 months were excluded from the study.

### 2.5. Methods of Data Collection

The data were gathered using structured questionnaires, which comprised (1) sociodemographic characteristics of the study participants—these data were supported by laboratory-based findings (parasitological methods)—and (2) the associated risk factors for parasitic infection in children. The questionnaires were prepared in English and further translated into Afan Oromo to collect accurate data for this study.

### 2.6. Assessment of Malnutrition

The assessment of malnutrition was conducted according to Deka and Kalita [15]. Standard anthropometric methods were used to assess the nutritional status of all children. A child's weight was recorded accurately in kilograms (kg). The accuracy of weighing was checked regularly, and zero errors were noted and corrected before individual weight measurements were taken. The recumbent length was measured in an infant meter for babies less than 2 years old. For children older than 2 years, the standing height was measured against the wall with the help of a measuring tape corrected to the nearest 1 cm. The mid-arm circumference was also measured by placing a measuring tape firmly (without compressing the tissues) around the left upper arm at a point midway between the tip of the olecranon process and the tip of the acromion.

### 2.7. Anthropometric Assessment, Nutritional Status Determination, and Grading

In this study, Gomez's system of classification of malnutrition was used. The child's weight was compared with that of a normal child (50^th^ percentile) of the same age. The weight of each participant was measured with a digital weighing balance to the nearest 0.1 kg. The measurements were done twice, and the average was taken to minimize error [16]. Grading was determined: mild malnutrition = 75–90% weight for age, moderate malnutrition = 60–74% weight for age, severe malnutrition < 60%. The percentage of reference weight for age was calculated as follows:

 $$\begin{array}{c} \%\text{reference weight for age}=\frac{\text{patient weight}\times 100}{\text{weight of normal child of the same age}}. \end{array}$$

### 2.8. Stool Sample Collection and Parasitological Methods

#### 2.8.1. Stool Sample Collection

The parents/guardians were advised to collect the child's fresh stool sample in a clean, prelabeled, wide-mouthed, container with a tight lid. The specimen was then processed within half an hour of collection. The color, consistency, and odor of the stool were noted. A naked-eye examination of stool was done to check for the presence of protozoal and parasitic (helminths) elements. Later, the stool samples were subjected to microscopic examination following the three parasitological methods described as follows.

#### 2.8.2. Wet–Mount

A direct wet–mount of stool specimen was conducted according to Cheesbrough [17]. A stool specimen was obtained from all study participants. Then, a direct saline wet–mount microscopy of each sample was used to detect intestinal protozoa and parasites (helminths) microscopically. In this method, one drop of normal saline was added on a clean slide, and then, a stool equivalent to a matchstick head (2 mg) was mixed with it. The stool and saline suspensions were examined under the light microscope under 100X and 400X magnifications to detect the structures of the parasites.

#### 2.8.3. Formalin–Ether Concentration

Formalin–ether concentration was conducted as described by Cheesbrough's [17] method. In this method, a portion of each preserved stool specimen was taken using an applicator stick and processed following the standard procedures. Briefly, 1 g of stool sample was placed in a clean conical centrifuge tube containing 7 mL of 10% formalin using an applicator stick. The resulting suspension was then filtered through a sieve into another conical tube. After adding 3 mL of diethyl ether to the solution, the content was centrifuged at 3200 rpm for 1 min. The supernatant was then discarded; the smear was prepared from the sediment and observed under a light microscope with a magnification of 10X and 40X.

#### 2.8.4. Modified Ziehl–Neelsen Method

Suspected samples for opportunistic protozoa were analyzed using the modified Ziehl–Neelsen method as described by Tahvildar-Biderouni and Salehi's [18] method. Smears were prepared from the concentrated stool samples, air dried, and fixed by ethanol. Later, alkaline fusion was poured on the slides and heated until it was brought up to steam but not boiled. After 5 min, the slides were washed with water and decolorized by 2.5% sulfuric acid for 1 min, depending on the film thickness, then counterstained with 1% methylene blue for 1 min, then washed and air dried, and examined with 100X magnification.

### 2.9. Identification of Parasites

The identification of parasites was performed based on the direct detection of the respective causative agent in clinical specimens using a compound microscope. The genus and/or species determination of the parasite was done by experienced laboratory technicians working in the hospital. In this study, all the developmental stages of the parasites (trophozoite, cyst, egg, larvae, and adult) were recorded. Examination (macroscopic and microscopic) of all specimens was performed according to Lynne [19].

## 3. Data Analysis

In this study, the data was analyzed using SPSS software version 26. The obtained values were considered significant at *p* < 0.05 in all the analyses. In addition, univariate and multivariate logistic regression analyses were considered to identify significant risk factors for parasitic infection among the study participants. The comparison among the proportions was also analyzed using the chi-square test (MedCalc Software Ltd., Comparison of Proportions Calculator).

## 4. Quality Control

In order to be certain of the quality of the obtained data, all the positive specimens were re-examined, and 10% of the negative specimens were randomly selected and re-examined by experienced laboratory technicians who did not have any information about the previous results. In addition, for quality checkup purpose, the control slides were also used during each run of the stain examination.

## 5. Results

### 5.1. Sociodemographic Characteristics of the Study Participants

In this study, a total of 422 children aged ≤ 14 participated, of which 50% were malnourished and the other 50 were well nourished in their nutritional status. Of the total study participants, 180 (42.65%) were males, and 242 (57.34%) were females (Table 1). The majority of the participants (56.39%) had a family size that varied from 4 to 7. On the other hand, the educational level indicated that the majority of the children were attending kindergarten (65.40%) and were followed by the illiterate ones (33.60%). A total of 96% of mothers' occupations were noted as housewife, and fathers' occupations were noted as farmer. The majority of the study participants were from the rural area (69.0%) in their residency. Interestingly, no nongovernmental organizations (NGOs) working with study participants' mothers and fathers were found in the present study (Table 1).

### 5.2. Trends of Parasitic Infection in Malnourished and Nonmalnourished Children

In this study, the status of parasitic infection was assessed among children with the characteristics of undernutrition and nonmalnourished groups in Bele Gesgar Primary Hospital. The overall infection of the parasites was 44.07% and 19.43% in malnourished and well-nourished children, respectively (Table 2). In both groups, the major intestinal parasite observed was *Entamoeba histolytica*/*dispar*, followed by *Giardia lamblia*, *Ascaris lumbricoides*, and hookworm (i.e., *Ancylostoma* spp.).

### 5.3. Status of Coinfection in Malnourished and Well-Nourished Children

This study also showed the existence of double and triple infections among the study participants. Among the double infection, the co-occurrence of *E. histolytica*/*dispar*+*G. lamblia* was observed to be the highest, showing no significant difference between the malnourished and nonmalnourished children (*p* > 0.05) as shown below (Table 2). In addition, the triple infection (*A. lumbricoides*+*H. nana*+*Taenia* spp.) between the malnourished and nonmalnourished children was found to be insignificant. The data of coinfection indicated the highest level of infection in females as compared to males. However, the middle-age class was the most infected group with protozoal and parasitic (helminths) coinfections as compared to the other two age groups.

### 5.4. Univariate and Multivariate Analyses of Contributing Factors for IPIs in Malnourished Children

The most important risk factor for parasitic infection among malnourished children attending Bele Gesgar Hospital was identified using univariate and multivariate logistic regression analyses (Table 3). The univariate and multivariate logistic regression analyses were conducted to evaluate the possible risk factors that are responsible for the intestinal protozoa parasites among and/or between the study participants. The analysis indicated that having no toilet (HT), not washing hands with soap after toilet (WHWSAT), having contact with pet animals (CWA), and playing with mud and soil (PWMS) were the significant risk factors of IPIs (*p* < 0.05).

### 5.5. Status of IPIs in Malnourished Children Based on Gender

In the present study, the tendency of parasitic infection was analyzed based on the sex of the study participants (Table 4). IPIs showed higher in females (60.22%) than males (39.78%) with a significant difference in statistics (*p* < 0.05). However, none of each parasite showed a significant difference between the two genders (*p* > 0.05).

### 5.6. Status of Parasitic Infection in Malnourished Children Based on Age

The trends of parasitic infection were investigated across age among the malnourished children who participated in this study. In this concern, the result indicated that the prevalence of the infection was found to be decreased as the age of the children increased (Table 5). High prevalence was observed in the age groups of ≤ 5, and the lowest was observed in the age groups of 11–14. The age groups that lie between 6 and 10 years old showed double and triple infections. The chi-square analysis revealed that both *Entamoeba histolytica*/*dispar* and *Giardia lamblia* showed significant differences based on the age-wise analysis. Overall, the total parasite infection based on age-wise analysis came with a significant difference (*p* < 0.05).

### 5.7. Intensity, Types, and Number of Parasite Infection in Malnourished Children

In Table 6, light/mild malnutrition was the most at 53.08%, followed by moderate at 43.60% and severe at 3.31%.

Of these, stunting malnutrition was demonstrated the highest of 45.5%, which differed significantly (*p* < 0.05) from stunting (26.06%) and underweight malnutrition (28.43%).

## 6. Discussion

Intestinal parasitic infections and undernutrition have continued to be major public health problems in Ethiopia, especially among children of various age groups. Thus, assessing IPIs and nutritional status of children is essential for improving the health conditions and academic performance through implementing appropriate intervention strategies [20]. Therefore, the present study was conducted to assess the magnitude of IPIs in malnourished children, compare them with those of nonmalnourished children, and determine the associated risk factors among children. In this study, 11 parasites were detected, namely, *Entamoeba histolytica*/*dispar*, *Giardia lamblia*, *Ascaris lumbricoides*, hookworm (*Ancylostoma* spp.), *Taenia* spp., *S. mansoni*, *Trichuris trichiura*, *Enterobius vermicularis*, *Hymenolepis nana*, *Cryptosporidium* spp., and *Cystoisospora belli.* Among the detected parasites, *Entamoeba histolytica*/*dispar* was the major parasite observed in malnourished children, showing a significant difference with nonmalnourished children (*p* < 0.05). The current study showed no difference between the two study groups except for the *Entamoeba histolytica/dispar*, which showed a significant difference (*p* < 0.05), showing that it is the most prevalent protozoa. This is in line with the study conducted by Bisetegn et al. [11] and a study conducted in Iraq that reported *E. histolytica* as the most common child-infecting protozoa, followed by *G. lamblia*, *H. nana*, and *E. vermicularis* [21]. The highest prevalence of *E. histolytica/dispar* may indicate the fecal contamination of foods and drinks and poor hygienic practice in the community. On the other hand, the predominance of *E. histolytica/dispar* infection might be associated with poor hygiene, poverty, lack of access to portable water, and a hot, humid tropical climate. Furthermore, their infective stage (the cyst) can withstand a standard level of chlorine treatment in drinking water [22].

The total parasite infection was higher in malnourished children as compared to well-nourished children, showing a statistically significant difference (*p* < 0.05). This indicated that the parasitic infection is more prevalent in undernourished children, and the nutritional status of a child is the best indicator of the child's overall health conditions [23]. IPIs harm the nutritional status of the infected individuals, and they may trigger bleeding of the gastrointestinal tract and nutrient competition, which may also lead to nutrient malabsorption [24]. Children with malnutrition and compromised immune systems are more susceptible to parasitic infections, which further weaken the immune function, perpetuate the cycle of infection, and increase malnutrition [25].

In addition, the overall prevalence of IPIs was variable among the studies conducted by different researchers in different countries. For example, our recent result of IPIs in undernourished children (44.07%) is lower than the study conducted by Onyemelukwe et al. [24], which was 51.8%, and it is higher than previous studies in Guinea (27%) among the malnourished children [26] but lower than 58.3% in Oscoda Lagos [23], 67.4% in Akwa Ibom Nigeria [27], and 52.4% in Ethiopia [20]. This difference might be due to environmental and geographical variation, differences in the study seasons, sanitation, hygienic practice, implementation of deworming, and poor supervision of children. Moreover, taking repeated stool samples could increase the chance of recovering multiple intestinal parasites.

This study also showed the existence of double infection and triple infection of parasites between the two studied groups (i.e., malnourished and well-nourished children). The study indicated that the prevalence of coinfection was higher in malnourished children as compared to the well-nourished children. This might be associated with different factors that may exist between the study participants. Environmental conditions could have an impact on host physiology and susceptibility to intestinal parasitic infections. For example, a stressed or malnourished host is more likely to be infected. In addition, host life history traits such as growth rate, lifespan, and fecundity have also been shown to favor coinfection in humans [28]. Interestingly, we found a low status of coinfection as compared to the study conducted by Zhang et al. [29] in southwest China. This might be due to the variations of the study area and methods employed in the detection of the parasites and the level of expertise in parasite identification. In addition, coinfections could either have synergetic or antagonistic effects on children's immune systems. For instance, children coinfected with hookworm and *Trichuris* have been shown to be more likely to have blood hemoglobin levels indicative of anemia than children harboring only one of these parasites [30]. Furthermore, coinfection may create opportunities for viral and bacterial infections among the children as the immune systems of the children may reduce due to intestinal parasitic (protozoa and helminths) infection.

In this study, females were found to be heavily infected by more than one parasite as compared to males. This is in agreement with a study conducted by Asa et al. [31], where double and triple infections were common and more prevalent among females. The overall prevalence of parasite infection was found to be significant between females and males of malnourished children. In addition, this study investigated the trends of parasitic infection based on the study participants' gender among the malnourished children. Thus, the study indicated a higher prevalence of parasitic infection in females, showing statistically significant variation as compared to the males (37% vs. 56%). This does not agree with a study in Iran [25] and in some studies in Ethiopia [22] that reported a higher prevalence of IPIs among males. However, this result was similar to the results from a study carried out in the southwest of Ethiopia, where the prevalence rate in females was slightly higher than in males [10]. The difference might be associated with the overall performance and/or activities performed by males and females in the country. In Ethiopia, male children are not mostly involved in caring for their elders and keeping, washing, and changing clothes (diapers). In addition, male children are not forced to clean houses and compounds and wash any dirty materials and baby's clothes. So this might be the cause for the reduction of parasite infection in males as compared to females in this study.

The significant risk factors (*p* < 0.05) for IPIs in this study were HT, not WHWSAT, having CWA, and PWMS. The absence of or not using toilets is believed to expose individuals to parasite infection. Open-air defecation is strongly associated with IPIs [28]. Similarly, studies have linked open-field defecation to the spread of intestinal parasite diseases [32, 33], because helminth ova and intestinal protozoan cyst stages can survive for a long time. As a result, children can become infected through a variety of routes.

The age-wise analysis between age increment and parasite infection seems to have an inverse relation to each other. The overall infection observed was 46.24%, 39.78%, and 13.98% for the age groups of < 5, 6–10, and 11–14 years old, respectively. This indicates that the lower age groups were more susceptible and the higher age groups were less susceptible for parasitic infection among the malnourished children. The lower age groups (< 5 years old) mostly play with soil and dirty materials that are found in their area; especially in this age group, some may use diapers and put anything from their surroundings into their mouth. Therefore, this may increase the probability of being infected by any parasites that contaminate those materials. In addition, these age groups have less information about parasites, self-cleaning, and the risk of parasitic infection, and thus, they pay less attention to such conditions, unlike the higher age groups. The older children are more likely to practice better personal hygiene than the younger ones, especially in the presence of food shortages [34]. Young children and females in impoverished areas are particularly vulnerable due to underdeveloped immune systems, poor hygiene, and gender-specific roles that increase exposure to contaminated environments [25]. Furthermore, the double and triple infections were mainly observed in the middle-age (i.e., 6–10 years) groups. Children in this group have less maturity as compared to the last age group (i.e., 11–14 years) and spend their time outside of home paying less care for them. So this might be the cause of the increased occurrence of coinfection in this age group.

Child malnutrition, which includes both under- and overnutrition, is a global health issue. The three primary AIs used to measure child's nutritional status are stunting, wasting, and underweight [35]. Underweight is a direct indicator of both chronic and acute malnutrition, since it represents both low height for age and low weight for age. Thus, in this study, we investigated the intensity of undernutrition among children based on three indicators (mild/slight, moderate, and severe). According to these indicators, 53.08% and 3.31% were mild/slight and severe, respectively. This may indicate that most of the children studied were lightly/mildly malnourished, and few of them were severely malnourished.

### 6.1. Limitations of the Study

There were some limitations in this study. Stool samples were collected only once, which may have reduced the number of protozoa and parasites (helminths) identified in this study. It is likely that the analysis of three consecutive stool samples could increase the number of protozoa and helminths to be identified in the study. There is a possibility that some of the participants might take antiparasitic drugs prior to stool sampling since metronidazole, mebendazole, and albendazole can be purchased without prescription in Ethiopia, which can even reduce the number of positive stool specimens. The prevalence of some protozoa and parasites (helminths) could be higher if culture techniques and quantification had been used. Quantification can determine whether children with higher parasite burdens have worse physical conditions. In addition, using coproparasitological techniques only instead of molecular methods might result in false-positive or negative findings. The variability of parasite infection in this study decreased as the laboratory work was not consistently performed by different individuals.

## 7. Conclusion

IPIs in this study area showed a high prevalence of 31.75% in malnourished children aged < 14 years, which is significantly higher than well-nourished children. The infection was high in females, unlike in males of malnourished children. In addition, no toilet usage, not WHWSAT, having CWA, and PWMS were found to be significant risk factors (*p* < 0.05) for IPIs in malnourished children. Hence, awareness-creation training should be given to Bele Gesgar areas to minimize parasite infections, and health offices should pay attention on the proper preparation and utilization of toilets. Parents should follow and take care of their children while they are staying out of home.

## Figures and Tables

**Table 1:** Sociodemographic characteristics of the study participants (*N* = 422).

**Variable**	**Category**	**Frequencies**	**%**
Sex	Male	180	42.65
	Female	242	57.35

Age	Below 5	197	46.70
	6–10	221	52.40
	11–14	4	0.90

Family size	1–3	125	29.0.62
	4–7	238	56.40
	Above 8	59	13.98

Educational level of children	Illiterate	142	33.65
	Kindergarten	276	65.40
	Grade 5–9	4	0.95

Mother's occupation	Government employee	16	3.80
	House wife	405	96.0
	Private business owner	1	0.20
	Nongovernmental organization	0	0.0

Father's occupation	Government employee	3	0.70
	Farmer	381	90.30
	Private business owner	38	9.0
	Nongovernmental organization	0	0.0

Residence	Urban	131	31.0
	Rural	291	69.0

**Table 2:** Comparisons of parasitic infection in malnourished (*n* = 211) and nonmalnourished children (*n* = 211).

**Identified parasite**	**No. of IPIs children (%)**	**No. of malnourished children (%)**	**No. of nonmalnourished children (%)**	**p** **-value**
Protozoa	—	—	—	—
*Entamoeba histolytica*/*dispar*	36	24 (11.37)	12 (5.69)	0.037⁣^∗^
*Giardia lamblia*	30	20 (9.48)	10 (4.74)	0.058
*Cryptosporidium* spp.	8	5 (2.37)	3 (1.42)	0.475
*Cystoisospora belli*	3	3 (1.42)	0 (0.0)	0.083
Helminths	—	—	—	—
*Ascaris lumbricoides*	12	8 (3.79)	4 (1.89)	0.241
*Taenia* spp.	5	4 (1.89)	1 (0.47)	0.177
Hookworm (*Ancylostoma* spp.)	10	6 (2.84)	4 (1.89)	0.521
*Schistosoma mansoni*	7	5 (2.37)	2 (0.95)	0.254
*Trichuris trichiura*	7	5 (2.37)	2 (0.95)	0.254
*Enterobius vermicularis*	1	1 (0.47)	0 (0.0)	0.319
*Hymenolepis nana*	5	3 (1.42)	2 (0.95)	0.656
Double infection	—	—	—	—
*E. histolytica*/*dispar*+*G. lamblia*	5	4 (1.89)	1 (0.47)	0.177
*Taenia* spp.+*G*. *lamblia*	2	2 (0.95)	0 (0.0)	0.156
*T*. *trichiura+S. mansoni*	1	1 (0.47)	0 (0.0)	0.319
Triple infection	—	—	—	—
*A. lumbricoides+H. nana*+*Taenia* spp.	2	2 (0.95)	0 (0.0)	0.156

Total	**134 (31.75)**	**93 (44.07)**	**41 (19.43)**	**p** < 0.0001^∗^

⁣^∗^Significant difference in statistic (*p* < 0.05).

**Table 3:** Regression (univariate and multivariate) analysis of associated risk factors for parasitic infection among malnourished children (*N* = 93).

**Items**		**No. of IPIs (%)**	**Logistic analysis**					
			**Univariate**			**Multivariate**		
			**COR**	**[CI]**	**p** **-value**	**AOR**	**[CI]**	**p** **-value**
HT	Yes	25 (26.88)	8.73	[4.7–16.3]	0.001	3.541	[1.2–10.50]	0.023⁣^∗^
	No	68 (73.12)	Ref.	—	—	—	—	—

WHWSAT	Yes	15 (16.13)	9.40	[4.8–18.4]	0.001	3.074	[1.3–7.30]	0.010⁣^∗^
	No	78 (83.87)	Ref.	—	—	—	—	—

WHWSBE	Yes	21 (22.58)	2.61	[1.42–4.8]	0.002	0.529	[0.2–1.50]	0.229
	No	72 (77.42)	Ref.	—	—	—	—	—

CWA	Yes	60 (64.52)	Ref.	—	—	—	—	—
	No	33 (35.48)	1.69	[0.10–3.0]	0.062	0.095	[0.03–0.32]	0.001⁣^∗^

ERFV	Yes	35 (37.63)	4.9	[2.70–8.8]	0.001	1.873	[0.5–7.30]	0.367
	No	58 (62.37)	Ref.	—	—	—	—	—

PWMS	Yes	81 (87.09)	Ref.	—	—	—	—	—
	No	12 (12.90)	15.4	[7.5–31.6]	0.001	13.210	[4.5–38.00]	0.001⁣^∗^

SDW	Packed	35 (37.63)	Ref.	—	—	—	—	—
	Pipe	20 (21.51)	0.927	[0.4–1.8]	0.823	1.308	[0.5–3.70]	0.616
	River	38 (40.87)	0.336	[1.2–3.5]	0.993	10.71	[1.4–2.10]	0.997

Abbreviations: aOR, adjusted odd ratios; CI, confidence interval; cOR, crude odd ratios; CWA, contact with animals; ERFV, eating raw fruits and vegetables; HT, having no toilet; IPIs, intestinal parasitic infections; No., number; PWMS, playing with mud and soil; Ref., reference; SDW, sources of drinking water; WHWSAT, washing hand with soap after toilet; WHWSBE, washing hand with soap before eating.

⁣^∗^Significant difference in statistic (*p* < 0.05).

**Table 4:** An analysis of intestinal parasitic infection among malnourished children based on sex (*n* = 93) using a chi-square (*χ*^2^**)** test.

**Parasites**	**No. of IPIs in gender (%)**		**χ** ^2^	**p** **-value**
	**Male**	**Female**		
Protozoa	—	—		—
*Entamoeba histolytica*/*dispar*	10 (10.75)	14 (15.05)	0.761	0.383
*Giardia lamblia*	8 (8.60)	12 (12.90)	0.891	0.345
*Cryptosporidium* spp.	2 (2.15)	3 (3.22)	0.203	0.652
*Cystoisospora belli*	0 (0.0)	1 (1.07)	0.995	0.319
Helminths	—	—		—
*Ascaris lumbricoides*	3 (3.22)	5 (5.38)	0.524	0.469
*Taenia* spp.	1 (1.08)	3 (3.22)	1.007	0.316
Hookworm (*Ancylostoma* spp.)	2 (2.15)	4 (4.30)	0.685	0.408
*Schistosoma mansoni*	2 (2.15)	4 (4.30)	0.685	0.408
*Trichuris trichiura*	4 (4.30)	2 (2.15)	0.685	0.408
*Enterobius vermicularis*	1 (1.08)	0 (0.00)	1.004	0.316
*Hymenolepis nana*	1 (1.08)	2 (2.15)	0.333	0.564
Double infection	—	—		—
*E. histolytica*/*dispar*+*G. lamblia*	2 (2.15)	2 (2.15)	0.00	1.000
*Taenia* spp.+*G*. *Lamblia*	1 (1.08)	1 (1.08)	0.00	1.000
*T*. *trichiura+S. mansoni*	0 (0.0)	1 (1.08)	1.004	0.316
Triple infection	—	—		—
*A. lumbricoides+H. nana*+*Taenia* spp.	0 (0.0)	2 (2.15)	2.010	0.156

Total	**37 (39.78)**	**56 (60.22)**	**7.729**	**0.005 **⁣^∗^

*Note: χ*2: chi-square test.

⁣^∗^Significant difference in statistic (*p* < 0.05).

**Table 5:** Intestinal parasitic infections among malnourished children based on age (*n* = 93).

**Parasites**	**No. of infected children in age groups (%)**			**χ** ^2^	**p** **-value**
	**≤ 5**	**6–10**	**11–14**		
Protozoa	—	—	—	—	—
*Entamoeba histolytica*/*dispar*	13 (13.98)	7 (7.53)	3 (3.23)	6.609	0.037⁣^∗^
*Giardia lamblia*	12 (12.90)	8 (8.60)	2 (2.15)	6.909	0.032⁣^∗^
*Cryptosporidium* spp.	2 (2.15)	3 (3.23)	0 (0.0)	2.800	0.24
*Cystoisospora belli*	1 (1.10)	1 (1.10)	0 (0.0)	ND	ND
Helminths					
*Ascaris lumbricoides*	5 (5.38)	2 (2.15)	1 (1.10)	3.250	0.197
*Taenia* spp.	0 (0.0)	2 (2.15)	2 (2.15)	2.000	0.368
Hookworm (*Ancylostoma* spp.)	3 (3.23)	2 (2.15)	1 (1.10)	1.000	0.607
*Schistosoma mansoni*	3 (3.23)	1 (1.10)	2 (2.15)	1.000	0.607
*Trichuris trichiura*	2 (2.15)	1 (1.10)	1 (1.10)	0.500	0.779
*Enterobius vermicularis*	0 (0.0)	1 (1.10)	0 (0.0)	ND	ND
*Hymenolepis nana*	0 (0.0)	3 (3.23)	0 (0.0)	6.000	0.050
Double infection					
*E. histolytica*/*dispar*+*G. lamblia*	2 (2.15)	2 (2.15)	0 (0.0)	2.000	0.368
*Taenia* spp.+*G*. *Lamblia*	0 (0.0)	2 (2.15)	0 (0.0)	ND	ND
*T*. *trichiura+S. mansoni*	0 (0.0)	1 (1.10)	0 (0.0)	ND	ND
Triple infection					
*A. lumbricoides+H. nana*+*Taenia* spp.	0 (0.0)	1 (1.10)	0 (0.0)	ND	ND

Total	**43 (46.24)**	**37 (39.78)**	**13 (13.98)**	**16.258**	**0.0003 **⁣^∗^

Total number of observations must be larger than 2. *χ*^2^: chi-square test.

⁣^∗^Significant difference in statistic (*p* < 0.05).

**Table 6:** Class intensity of malnutrition and types of malnutrition detected in malnourished children.

**Intensity and type of malnutrition**	**Categories**	**No. of IPIs (%)**	**Frequencies (%)**	**χ** ^2^	**p** **-value**
Intensity	Light/mild	45	112 (53.08)	88.39	0.001⁣^∗^
	Moderate	33	92 (43.60)		
	Severe	15	7 (3.31)		

Malnutrition	Stunting	38	55 (26.06)	14.22	0.001⁣^∗^
	Wasting	23	96 (45.5)		
	Underweight	32	60 (28.43)		

*Note:χ* ^2^: chi-square. No. of IPIs (%): total number of intestinal parasitic infections found in malnourished children in percentage.

⁣^∗^Significant difference in statistic (*p* < 0.05).

## Data Availability

The data used for this study will be available upon request from the corresponding author.

## Nomenclature

aOR: Adjusted odd ratios
AIs: Anthropometric indicators
CI: Confidence interval
cOR: Crude odd ratios
IPIs: Intestinal parasitic infections
NGO: Nongovernmental organization
SPSS: Statistical packages for social sciences
WHO: World health organization
